# Adenosine Monophosphate Forms Ordered Arrays in Multilamellar Lipid Matrices: Insights into Assembly of Nucleic Acid for Primitive Life

**DOI:** 10.1371/journal.pone.0062810

**Published:** 2013-05-07

**Authors:** Laura Toppozini, Hannah Dies, David W. Deamer, Maikel C. Rheinstädter

**Affiliations:** 1 Department of Physics and Astronomy, McMaster University, Hamilton, Ontario, Canada; 2 Origins Institute, McMaster University, Hamilton, Ontario, Canada; 3 Department of Biomolecular Engineering, University of California Santa Cruz, Santa Cruz, California, United States of America; 4 Canadian Neutron Beam Centre, National Research Council Canada, Chalk River Laboratories, Chalk River, Ontario, Canada; University of Quebect at Trois-Rivieres, Canada

## Abstract

A fundamental question of biology is how nucleic acids first assembled and then were incorporated into the earliest forms of cellular life 4 billion years ago. The polymerization of nucleotides is a condensation reaction in which phosphodiester bonds are formed. This reaction cannot occur in aqueous solutions, but guided polymerization in an anhydrous lipid environment could promote a non-enzymatic condensation reaction in which oligomers of single stranded nucleic acids are synthesized. We used X-ray scattering to investigate 5′-adenosine monophosphate (AMP) molecules captured in a multilamellar phospholipid matrix composed of dimyristoylphosphatidylcholine. Bragg peaks corresponding to the lateral organization of the confined AMP molecules were observed. Instead of forming a random array, the AMP molecules are highly entangled, with the phosphate and ribose groups in close proximity. This structure may facilitate polymerization of the nucleotides into RNA-like polymers.

## Introduction

Prior studies have shown that RNA-like polymers can be synthesized non-enzymatically from mononucleotides in conditions simulating a prebiotic hydrothermal site undergoing cyclic fluctuations in hydration [1]. The presence of a phospholipid such as phosphatidylcholine markedly enhanced the yield of polymeric products, presumably because the lipid matrix serves to concentrate and organize the mononucleotides. The idea of guided polymerization dates back more than forty years ago [2]–[4], but there have been no studies of the arrangement of monomers within an organizing matrix. The primary aim of the research reported here was to determine whether in fact mononucleotides are captured and organized within a multilamellar structure that is produced when liposomes and solutes undergo dehydration. The results also represent a critical test of the proposed mechanism by which mononucleotides polymerize within the matrix. If it cannot be demonstrated that mononucleotides are captured in layers between lipid lamellae, the hypothesis would be excluded as a possible explanation.

**Figure 1:**
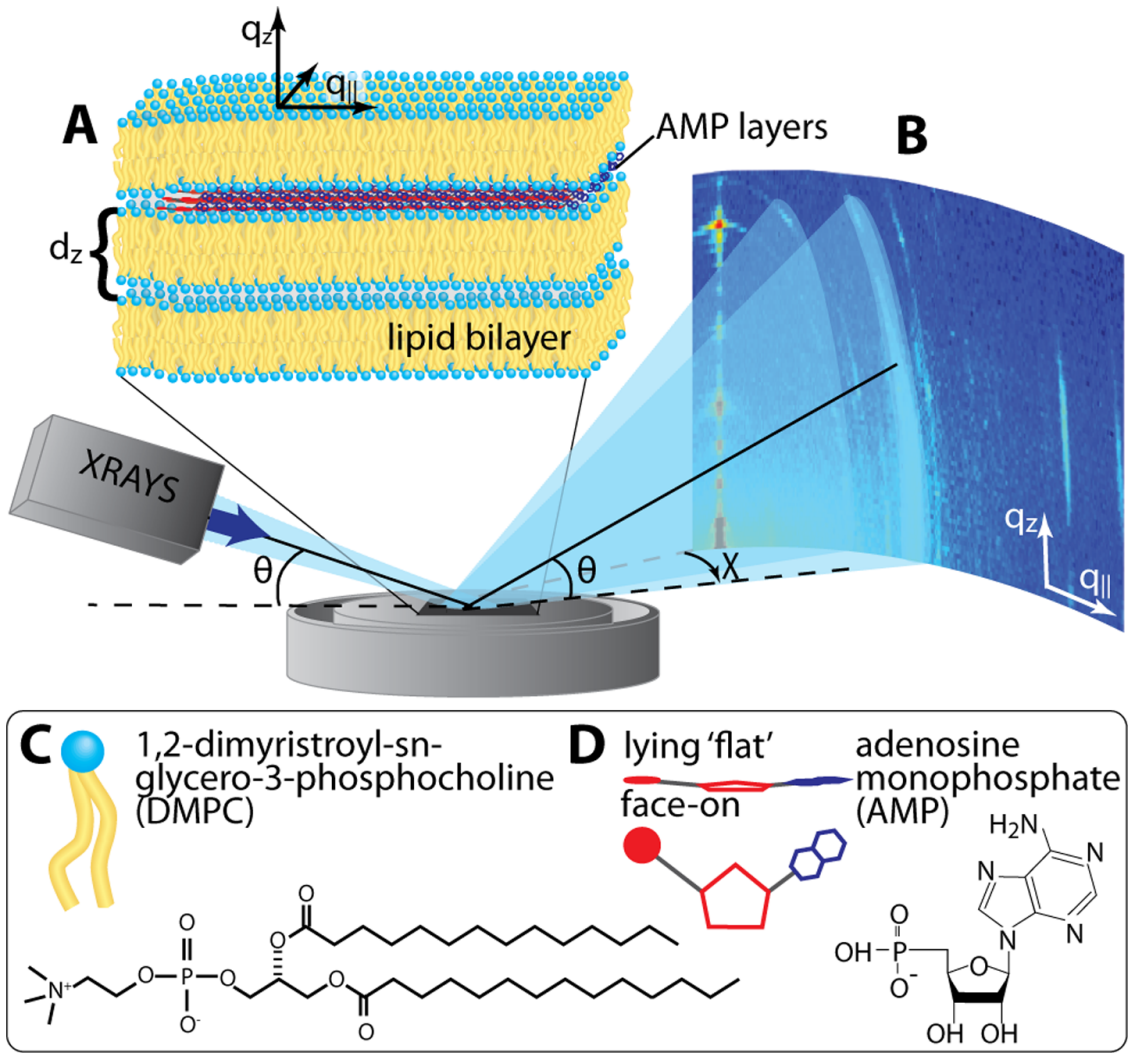
Schematic showing the experimental setup. **A** Lipids and AMP form a highly oriented multilamellar structure with the AMP molecules confined between the bilayers. **B** Diffraction of X-rays from the highly oriented solid supported lipid/AMP complexes. *q_z_* and *q_||_* are the out-of-plane and in-plane components of the scattering vector, *Q*. Chemical structures and representations of **C** dimyristoylphosphocholine (DMPC) and **D** adenosine monophosphate (AMP) molecules are depicted.

To test whether such organization occurs, we used X-ray scattering to investigate 5′-adenosine monophosphate (AMP) molecules captured in a multilamellar phospholipid matrix composed of 1,2-Dimyristoyl-sn-glycero-3-phosphocholine (DMPC), as shown in Figure 1. We chose AMP for this study because it was used in the earlier report that demonstrated its polymerization in a lipid matrix. Guanosine monophosphate (GMP) was not investigated because of its well known tendency to form relatively insoluble aggregates. The in-plane and out-of-plane structure of the bilayer/AMP complexes was determined with sub-nanometer resolution from these measurements, and Bragg peaks corresponding to the lateral organization of the confined AMP molecules were observed. Instead of forming a random array, or a geometrically favorable herringbone or chevron structure, the AMP molecules are highly entangled, with the phosphate and ribose groups in close proximity. This structure may facilitate polymerization of the nucleotides into RNA-like polymers.

## Results

**Figure 2:**
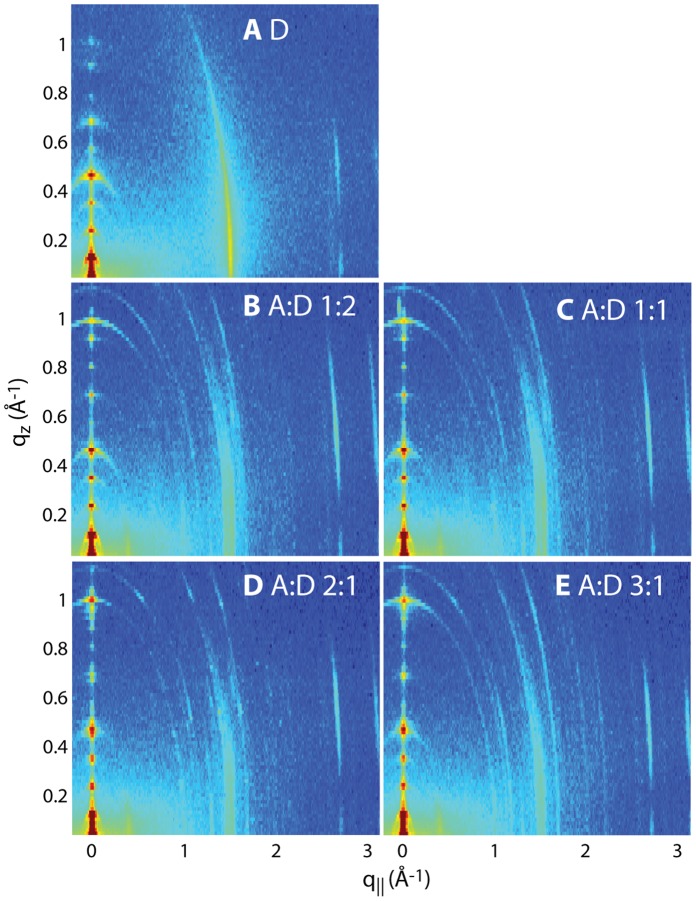
Two-dimensional X-ray intensity maps for all measured concentrations: **A** pure DMPC; **B** AMP:DMPC 1∶2; **C** AMP:DMPC 1∶1; **D** AMP:DMPC 2∶1; **E** AMP:DMPC 3∶1. All ratios are molar ratios. *q_z_* and *q_||_* are the out-of-plane and in-plane components of the scattering vector, *Q*.

Five different membrane complexes with different concentrations of AMP molecules were studied, as detailed in the Materials and Methods Section. Figure 2 shows 2-dimensional X-ray intensity maps for all measured concentrations: **A** pure DMPC (D), **B** AMP:DMPC 1∶2, **C** AMP:DMPC 1∶1, **D** AMP:DMPC 2∶1 and **E** AMP:DMPC 3∶1. The ratios are given as molar ratios. As depicted in Figure 1, the samples were oriented such that the *q_||_* axis probed lateral membrane structure and the perpendicular axis, *q_z_*, probed out-of-plane structure of the multilamellar membrane complexes. The data in Figure 2 cover a large area of reciprocal space and allow the molecular structure of the membrane/AMP complexes to be deduced.

The 100% DMPC sample in Figure 2 **A** shows a well developed in-plane Bragg peak along the *q_||_*-axis. The diffracted intensity has a distinct rod-like shape, indicative of a 2-dimensional system. The out-of-plane scattering along *q_z_* shows pronounced and equally spaced Bragg intensities due to the multi-lamellar structure of the membrane sample. The presence of AMP molecules in Figures 2 **B–E** leads to additional features along both out-of-plane (*q_z_*) and in-plane (*q_||_*) axes.

**Figure 3:**
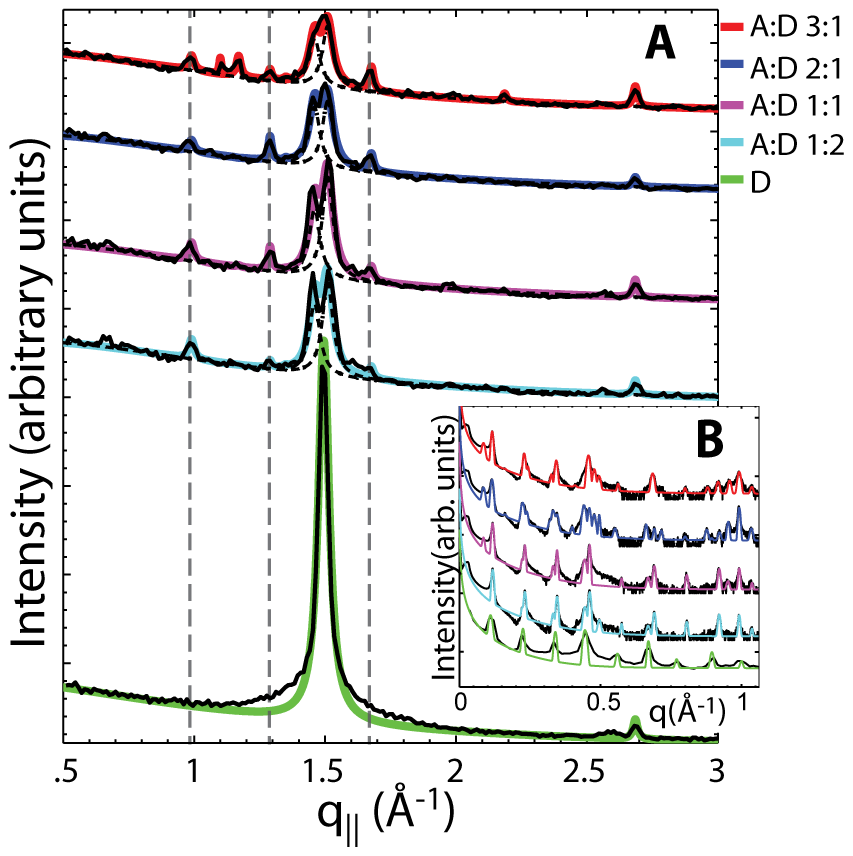
Projections of the 2-dimensional data in Figures 2 along the **A** in-plane and **B** out-of-plane axis. AMP:DMPC 3∶1 (red); AMP:DMPC 2∶1 (blue) AMP:DMPC 1∶1 (magenta); AMP:DMPC 1∶2 (cyan); DMPC only (green). The lateral arrangement of the AMP molecules was determined from the in-plane peaks in **A**. The distance between two bilayers in the stack is determined by the analysis of the out-of-plane reflections in **B**.

**Figure 4:**
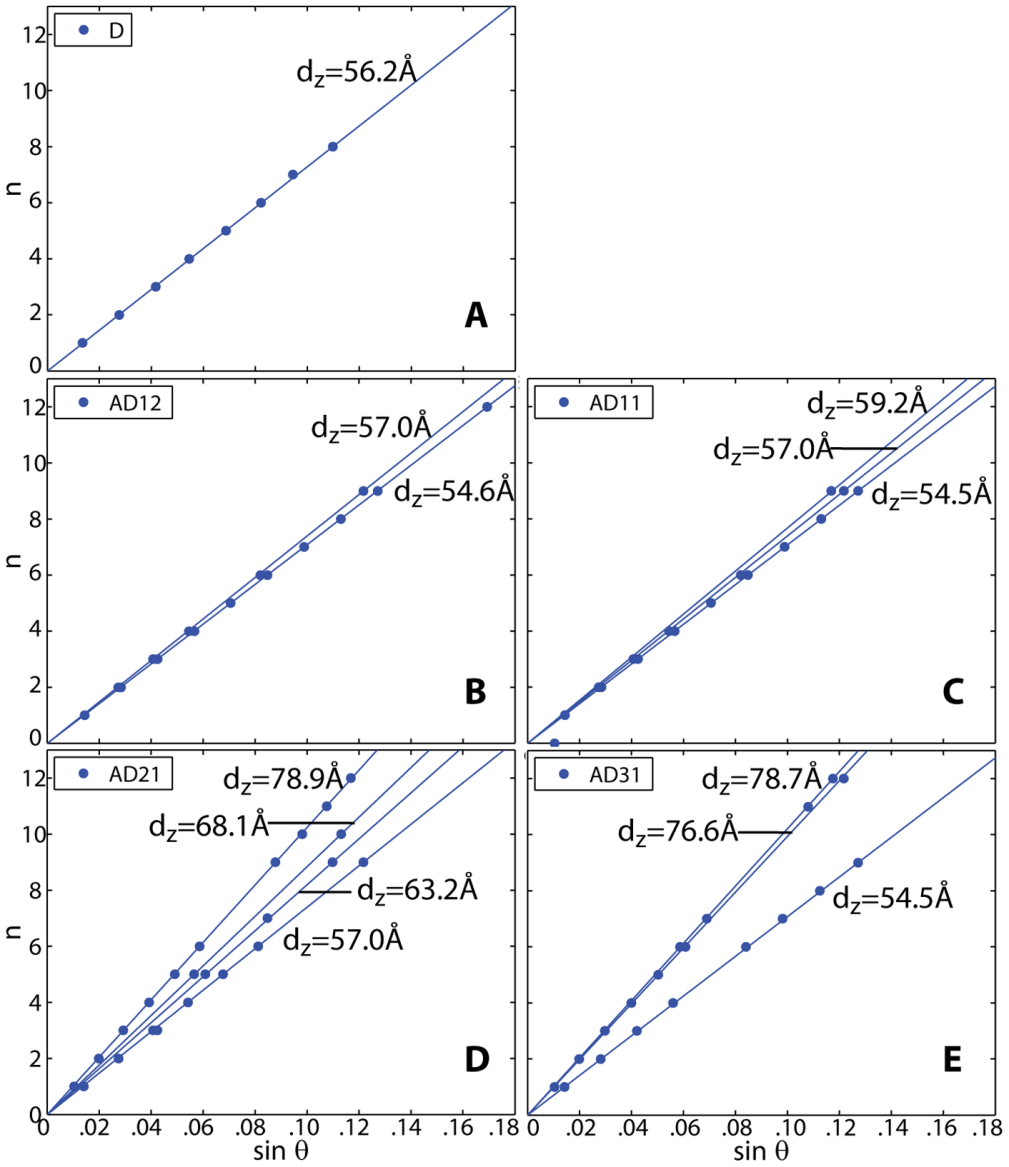
Peak order of peaks from the out-of-plane data in Figure 3 vs.sin(θ), where θ is the Bragg angle. The slope of each line shown is proportional to the distance between membranes in the stack, *d_z_* (*n = 2d_z_/λ sin(θ)*). **A** pure DMPC, **B** AMP:DMPC 1∶2, **C** AMP:DMPC 1∶1, **D** AMP:DMPC 2∶1, **E** AMP:DMPC 3∶1. Values for the different *d_z_* are given in the Figures.

**Figure 5:**
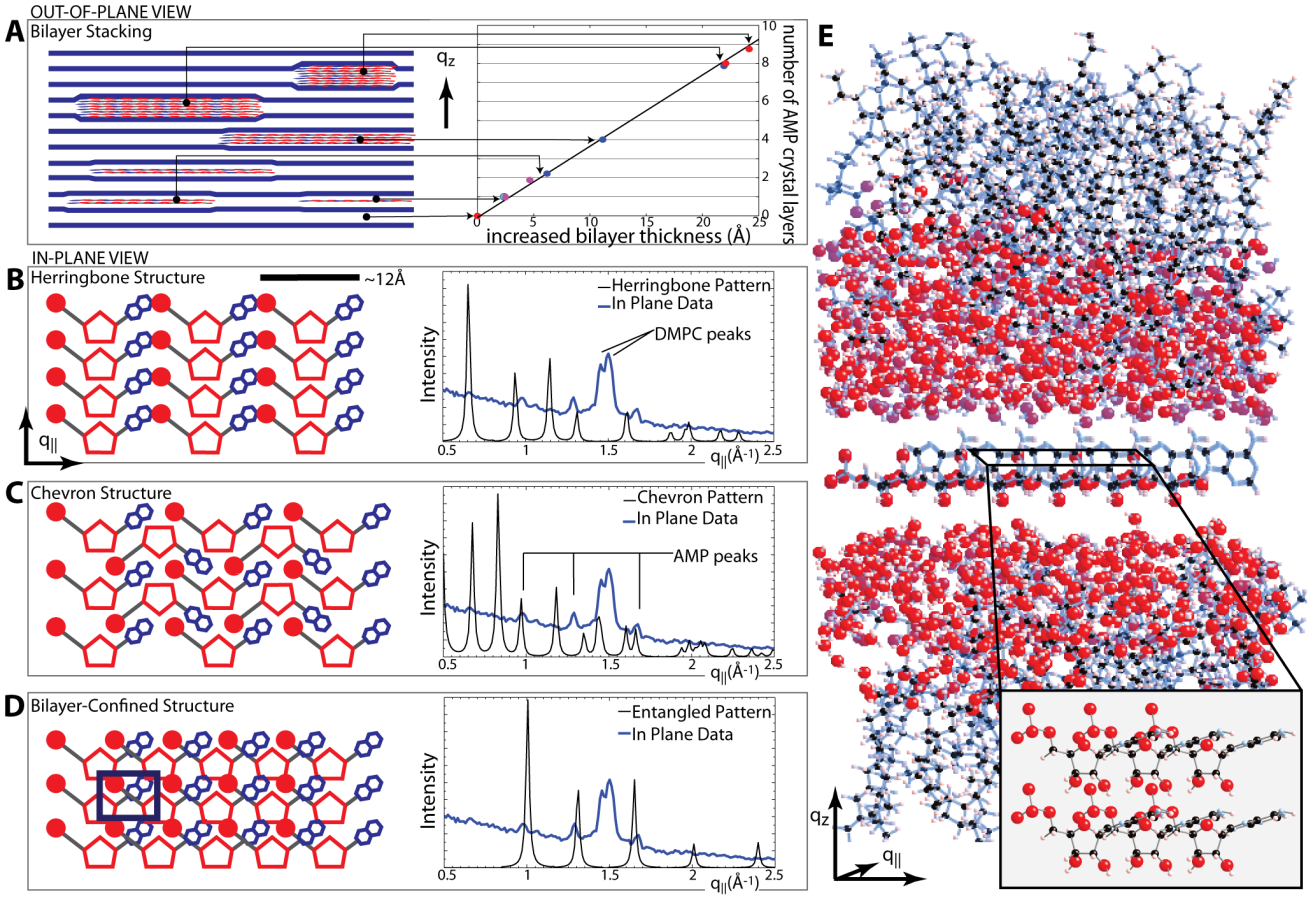
Crystal structure determination. **A** Schematic of out-of-plane structure of the lipid/AMP complex. The lamellar spacings determined in Figure 4 all fall on a master curve. The thickness of a single AMP layer, Δd, is determined by the slope to Δd = 2.67 Å. **B-D** show proposed AMP in-plane crystal structures and the resulting diffraction pattern (black) compared against the peak locations in our in-plane data (blue). **B** A herringbone structure would be geometrically favorable due to the shape of AMP. The diffraction pattern, however, does not agree with our experimental data. **C** The chevron pattern is also a favorable structure for the packing of ’v’ shaped molecules, however the diffraction pattern that would be produced from this structure is not consistent with the experiential data. **D** The tetragonal 2-dimensional unit cell with lattice parameters of a = 6.25 Å and b = 4.8 Å gives the most plausible structure of the AMP molecules. **E** Molecular representation of the crystalline AMP between the stacked DMPC bilayers; in-plane representation below. The molecular coordinates for the DMPC bilayer were taken from [*29*]. Molecular structure files of the structures in **D** and **E** are provided in Structure Files S1 and S2.

For a quantitative analysis of the diffracted intensity, the 2-dimensional data were cut along the out-of-plane and in-plane axes. As in-plane features are usually orders of magnitude weaker than the pronounced out-of-plane reflections, slices 0.03 Å^−1^< *q_z_* <0.3 Å^−1^ were integrated to enhance the data quality. The results for all samples are shown in Figure 3 **A** and **B**. The lamellar spacings *d_z_* of the membrane/AMP complexes, i.e., the distance between two bilayers in the membrane stack, was determined from the specular reflectivity in Figure 3 b) and are shown in Figures 4**A**–**E**. Because the complexes were prepared by drying and fusion of unilamellar vesicles in an AMP solution [5], [6], two lipid bilayers enclose the AMP molecules from above and below. The AMP phase of the complex, therefore, coexists with a pure DMPC phase. The lowest slope of each plot was assigned to the pure DMPC bilayer spacing (*d_z_^lipid^*). Figure 4 **A** shows the lamellar spacing of a single component lipid bilayer. Two distinct *d_z_*-spacings are observed for AMP:DMPC 1∶2 in Figure 4 **B**. Figures 4 **C**, **D** and **E** feature up to four different slopes for the higher concentrated samples, AMP:DMPC 1∶1, AMP:DMPC 2∶1 and AMP:DMPC 3∶1. For each concentration, the increase in lamellar spacing in the presence of AMP molecules was compared to the *d_z_^lipid^* of the coexisting DMPC phase, *d_z_–d_z_^lipid^ = mΔd*, where *m* is an integer number. The result is plotted in Figure 5 **E**. The data points for all concentrations fall on the same line through the origin and are well fit by a *Δd* of *Δd* = 2.67 Å. We assign this increment to the thickness of an interstitial single layer of AMP molecules. *Δd* = 2.67 Å indicates that the quasi 2-dimensional AMP molecules take a flat position between the stacked bilayers.

The lateral arrangement of lipid and AMP molecules was determined from the in-plane data in Figure 3 **A**. A correlation peak is observed for pure DMPC (D) at a *q_||_* position of *q_||_* = 1.50 Å^−1^. This peak agrees well with the acyl chain correlation peak reported for DMPC in its gel phase [7] and corresponds to a distance of *a_nn_* = 4π/(sqrt(3)*q_||_*) = 4.84 Å between neighboring lipid tails.

The presence of AMP molecules in the lamellar structure had two effects on the lateral structure: (1) The lipid peak split into two peaks and (2) additional peaks were observed. The additional peaks are located at *q_||_* = 0.99 Å^−1^ (6.4 Å), 1.29 Å^−1^ (4.9 Å), and 1.68 Å^−1^ (3.7 Å). As these peaks are not present in the pure DMPC sample, we assigned these peaks to the lateral structure of the confined AMP molecules.

Each AMP-containing sample has two central peaks corresponding to an average distance between two lipid acyl tails in the bilayers of *q_||_* = 1.46 Å^−1^ (4.97 Å) and 1.51 Å^−1^ (4.80 Å). The smaller tail-spacing agrees well with the spacing determined in the pure DMPC bilayer and was, therefore, assigned to the DMPC phase of the complex. The second peak at slightly larger nearest neighbor distances was assigned to the AMP phase, i.e., to the regions of the bilayers interacting with the AMP molecules. The distance between two neighboring acyl chains was found to slightly increase when in contact with AMP molecules from 4.84 Å to 4.97 Å. This is most likely a sign of an interdigitated structure. The ratio between the pure lipid phase and the DMPC/AMP phase was determined from the ratio between the integrated intensities of the two peaks and is given in Table 1. As expected, this ratio is close to 1∶1 for all samples and basically a result of the way the lipid/AMP complexes were prepared.

**Table 1:** Molar concentrations, number of AMP molecules associated with each lipid, and number of AMP molecules in the inter-lamellar space between two lipids (assuming that approximately every second bilayer excludes AMP molecules).

AMP:DMPC	AMP/lipid	AMP/lipids inter-lamellar	Lipid/AMP Fraction	Area per lipid molecule (Å^2^)	# of layers calculated	# of layers observed
0∶1	0	0	–	40.8	–	–
1∶2	1	2	0.76∶1	40.0	1.4	1
1∶1	2	4	0.67∶1	40.0	2.8	1,2
2∶1	4	8	0.89∶1	40.0	5.9	2,4,8
3∶1	6	12	0.78∶1	40.0	8.5	8,9

The Lipid/AMP fraction refers to the ratio of lipid correlation peaks in Figure 3 **A**, normalized to the pure lipid peak. The positions of these lipid peaks were used to calculate the area per lipid molecule, which increased from 40.0 to 42.8 Å with AMP present. The area per AMP was calculated by multiplying the unit cell parameters of the tetragonal unit cell to 30.0 Å. The number of layers between a bilayer as obtained from calculation and from the data in Figure 4 is also included.

The area per lipid can be determined by assuming that the lipid tails form a densely packed structure with hexagonal symmetry (planar group P6). The lipid area is then determined from the position of the lipid acyl chain correlation peak to A_L_ = 16π^2^(sqrt(3)*q_T_^2^*) [8], [9].

## Discussion

Possible 2-dimensional structures and their corresponding diffraction patterns are depicted in Figure 5. The occurrence of a series of correlation peaks is indicative of a well ordered structure rather than a gas or fluid phase of molecules having positional and orientational disorder. The two most intuitive planar arrangements of kinked, ’v’-shaped 2-dimensional molecules are the herringbone (Figure 5 **B**) and the chevron structure (Figure 5 **C**). Both structures are described by a tetragonal planar space group. However, the calculated diffraction patterns do not agree with the observed pattern. The diffraction pattern observed in Figure 3 **A** indicates a more densely packed, entangled structure. The molecular arrangement of the AMP molecules, which is compatible with the experimental data, is shown in Figure 5 **D**: the AMP forms 2-dimensional crystalline patches with positional and orientational ordering of the molecules. The corresponding unit cell is rectangular with lattice parameters *a* = 6.25 Å and *b* = 4.8 Å. Calculated and measured patterns show excellent agreement within the resolution and statistics of this experiment. The absence of Bragg reflections other than those from the lamellar membrane structure in the out-of-plane data in Figure 4 indicates that the AMP crystallites consist of 2-dimensional ordered layers, which are randomly oriented along the perpendicular *z* direction.

This 2-dimensional structure of the confined AMP molecules is significantly different from the structure of crystalline AMP, which crystallizes in a less densely packed 3-dimensional monoclinic structure P2_1_ with unit cell dimensions of *a* = 12.77 Å, *b* = 11.82 Å, *c* = 4.882 Å and β = 92.24° [10].

The AMP pattern in Figure 5 **D** can also be validated by the observed increase in lamellar spacings. The ratio between the area per lipid and the area per AMP molecule from the in-plane data in Table 1 determines the maximum number of AMP molecules that can be hosted by one lipid molecule. When multiplied by the total number of AMP molecules per lipid molecule, the number of AMP layers can be calculated. The area per lipid in the AMP:DMPC 3∶1 sample for instance is determined to be 42.2 Å^2^; the area per AMP is 30.0 Å^2^. The ratio 30.0/42.2 = 0.71 is multiplied by the number of AMP molecules between the bilayers to receive the thickness of the AMP crystallite, 0.71×12 = 8.5. Experimentally, *d_z_*-spacings corresponding to 8 and 9 AMP layers were observed, as listed in Table 1, to be in excellent agreement. The more spacious herringbone and chevron structures with their larger areas per AMP molecule would lead to distinctly thicker AMP structures as the lipids can host fewer AMP molecules, in disagreement with the experimental observations.

**Figure 6:**
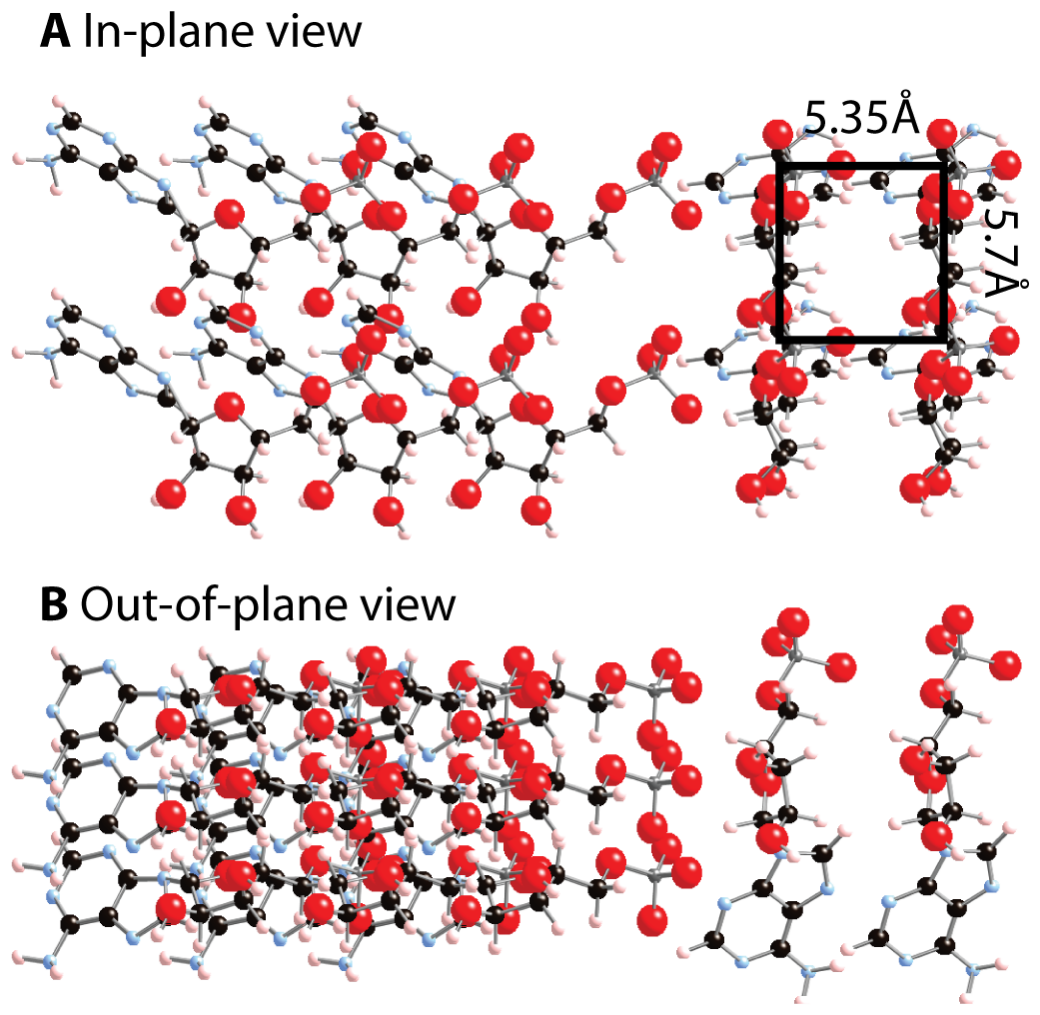
Structure in A:D 3∶1 bilayers. In the 3∶1 fit in Figure 3 **A**, the planar structure (as in all other AMP/DMPC samples) was found to coexist with another structure. The new crystal structure is compatible with AMP molecules oriented perpendicular to the existing structure. **A** The coexistence of two crystal structures as viewed in the plane of the bilayers. A unit cell is drawn for clarity. **B** Crystal structures as viewed from out of the plane of the lipid bilayers. A molecular structure file of the structures is provided in Structure File S3.

The positions of the in-plane correlation peaks did not change with increasing AMP concentrations, as indicated by the vertical lines in Figure 3**A**. The addition of more AMP molecules in more concentrated samples increases the thickness of the AMP layers but did not change the lateral structure. Additional peaks were observed at the highest concentration of AMP:DMPC 3∶1 at positions of *q_||_* = 1.1 Å^−1^, *q_||_* = 1.17 Å^−1^ and *q_||_* = 2.2 Å^−1^. The new peaks co-exist with the structural peaks from the entangled structure in Figure 5 **D** and can be described by a tetragonal unit cell with lattice parameters of *a* = 5.35 Å and *b* = 5.7 Å. The resulting structure is compatible with AMP molecules taking an upright position between the bilayers, as shown in Figure 6. This occurs when the thickness of the AMP crystallites becomes larger than the length of an AMP molecule of ∼17.5 Å. The concentration of AMP:DMPC 2∶1, therefore, is the maximum concentration at which the purely 2-dimensional AMP structure in Figure 5 **D** can be observed. The lowest concentration for which this entangled AMP structure was observed in the experiment was the AMP:DMPC 1∶2 ratio. This concentration was found to result in a single layer of AMP molecules between the membranes, as listed in Table 1. We can, however, not exclude that small ordered patches would form at even lower concentrations of AMP.

The pattern in 5 **D** brings the phosphate group in close proximity to the 3′ position of the ribose. The distance between the two groups (phosphorus and 3′ oxygen) can be estimated from the molecular structure to be ∼2.1 Å, approximately the length of a P-O bond [11]. Thus, the observed pattern of the confined AMP molecules may favor the formation of RNA-like structures when chemical binding is induced. When confined between the stacked membranes, the chemical potential provided by anhydrous conditions and elevated temperature may be sufficient to drive the synthesis of phosphodiester bonds between nucleoside monophosphates to form RNA-like structures [1], [12]–[14]. Furthermore, there is a growing consensus that self-assembled lipid membranes would have provided the compartments necessary to maintain systems of polymeric catalysts in the evolutionary pathway leading to the origin of cellular life [15]–[17].

### Conclusion

There is a consensus that a form of life based primarily on RNA likely preceded the RNA-DNA-protein world of the ancestral cell. However, in the absence of enzymes and metabolism there has been no obvious way for RNA-like molecules to be produced [18] and then encapsulated in cellular compartments, an essential first step in the origin of cellular life. Hydrothermal springs have been proposed as analogues of the prebiotic Earth [4], [19]. Cycles of hydration and dehydration at elevated temperatures can be used to simulate conditions in the neighborhood of volcanic hydrothermal springs. Rajamani *et al.* [1] showed that such conditions activate condensation reactions that can polymerize mononucleotides organized in a lipid matrix.

The present study adds significant weight to this model, because the pattern shown in 5 **D** brings the phosphate group of AMP in close proximity to the 3′ position of the ribose. The distance between the two groups can be estimated from the molecular structure to be ∼2.1 Å. The observed pattern of the confined AMP molecules may thus favor the formation of RNA-like polymers when the chemical potential provided by anhydrous conditions drives the synthesis of phosphodiester bonds between nucleoside monophosphates.

## Materials and Methods

### Preparation of the Supported Bilayer-AMP Complexes

Multi lamellar solid-supported lipid bilayers were prepared on single-side polished silicon wafers by vesicle fusion [20]–[22]. 300 µm thick Si(100)-wafers were pre-cut into 1×1 cm^2^ chips and cleaned by immersing the wafers in an H_2_O_2_/sulfuric acid mixture (volume fraction of 75% concentrated H_2_SO_4_, 25% H_2_O_2_ at 40°C for ∼1 hour). This strongly oxidizing combination removes all organic contaminants on the surface, but does not disturb the native silicon oxide layer. The wafers were then rinsed and stored under ultra pure water with a resistivity of 18.2 MΩ·cm before use [23].

Small lipid vesicles (liposomes) were prepared by dispersing 1,2-dimyristoyl-sn-glycero- 3-phoshatidylcholine (DMPC) in ultra pure water to produce concentrations of 10 mM and 20 mM. The milky solution, which initially contained multilamellar vesicles (MLVs), was sonicated for 15 minutes until the solution became transparent, indicating that small unilamellar vesicles (SUVs) formed. The free acid form of 5′-adenosine monophosphate (AMP) powder was added to ultra pure water in 10 mM and 20 mM concentrations and heated in a water bath until completely dissolved. The AMP solution and DMPC suspension were then mixed in molar ratios of (AMP:DMPC) 1∶2, 1∶1, 2∶1, and 3∶1.

A silicon chip was placed on a hot-plate and heated to 85°C. A 50 µL aliquot of the final suspension was pipetted onto the wafer, forming a 5 mm drop that completely dried in ∼1 minute. A control sample of pure DMPC was prepared from the 10 mM SUV dispersion. Care was taken to maintain the lipid and nucleotide solution at a temperature of at least 30°C during the deposition process and storage to keep the bilayers in their fluid phase above the phase transition temperature (T_m_) of 23.9°C [24]. By applying this procedure, small bilayer patches initially form on the substrate, which eventually undergo a transition into larger, more uniform layers. The sharp Bragg peaks in the X-ray experiment (see below) indicated that a highly oriented, multilamellar structure forms on the silicon wafer with a total thickness of ∼10 µm. Because the unilamellar vesicles and AMP solutions are prepared separately before being mixed, the interior of the vesicles does not contain any AMP molecules. During drying, the vesicles first form a concentrated gel on the silicon surface, then undergo further drying and fusion into multilamellar structures parallel to the plane of the silicon surface. Previous studies [5], [6] demonstrated that small solutes such as AMP are confined between alternating bilayers because the empty interiors of the vesicles exclude solutes during the fusion process. We envision that a typical structure consists of a thin layer of AMP molecules separated from the next AMP layer by two lipid bilayers (Figure 1).

### X-ray Scattering Experiment

X-ray scattering data was obtained using the Biological Large Angle Diffraction Experiment (BLADE) in the Laboratory for Membrane and Protein Dynamics at McMaster University. BLADE uses a 9 kW (45 kV, 200 mA) CuK rotating anode at a wavelength of 1.5418 Å. Both source and detector are mounted on movable arms such that the membranes stay horizontal during the measurements. Focusing multi-layer optics provides a high intensity parallel beam with monochromatic X-ray intensities up to 10^10^ counts/(mm^2^×s). This beam geometry provides optimal illumination of the solid supported membrane samples to maximize the scattering signal. A sketch of the scattering geometry is shown in Figure 1. Note that there is no risk of sample damage using this in-house technique because of the large beam size and relatively low intensity of the X-ray beam as compared to synchrotron sources.

By using highly oriented, solid-supported lipid bilayers, the in-plane (*q_||_*) and out-of-plane (*q_z_*) structure of the membranes was determined. From the high-resolution X-ray diffraction experiments we determine the molecular structure of the membranes in two different ways: (1) the out-of-plane X-ray data is used to determine the structure perpendicular to the membranes and (2) the lateral organization of the molecular components in the plane of the membrane, as sketched in Figure 1. The result of such an X-ray experiment is a 2-dimensional intensity map of a large area (0.03 Å^−1^< *q_z_* <1.2 Å^−1^ and 0.003 Å^−1^< *q_||_* <3.1 Å^−1^) of the reciprocal space, as sketched in Figure 1. All scans were measured at 20°C and 50% hydration, in the gel (L_β_) phase of the DMPC bilayers [25], [26]. Structural features are more pronounced in this state as fluctuations, which lead to attenuation and smearing of Bragg peaks, are suppressed. The sample was mounted in a so-called humidity chamber during the measurements in a saturated Mg(NO_3_)_2_ salt solution, which provided a relative humidity of 52.9%. The temperature was controlled using a circulating bath controller to 20°C with a stability of 0.1°C.

The lamellar spacing of the membrane AMP complexes was determined from the specular reflectivity. Figure 3 **B** shows out-of-plane data and fits for the five samples. Up to 24 Bragg peaks could be identified for a given sample and assigned to different *d_z_*-spacings and phases. The peaks were well fit by Gaussian peak profiles. To assign the peaks to different phases, Braggs law can be re-written as *n = 2d_z_/λ sin(θ)*. By plotting the order of the different Bragg reflections against the sine of the Bragg angles, *n* vs. *sin(θ(n))*, peaks which belong to the same *d_z_*-spacing fall on a straight line through the origin, whose slope is proportional to *d_z_*. The corresponding data are shown in Figures 4 **A–E**; up to a peak order n of 12 was observed. Not all diffraction orders are necessarily observed for the different *d_z_*-spacings as their scattering intensity depends on the form factor of the bilayers and oscillates between zero and maximum intensity as a function of *q_z_*.

We note that this experiment cannot be compared to protein crystallography, where atomic resolution protein structure is determined from protein crystals. The AMP molecules in our experiment are embedded in a lamellar membrane complex. The corresponding structure is inherently disordered, which leads to a strong suppression of higher order Bragg peaks in the experimental data. The organization of DNA molecules in liposomes/DNA complexes was reported using a similar technique [27]. The atomic structure of the AMP molecules was taken from [10]. The molecule was found to be very flexible, especially in the rotation of the adenine group such that the AMP molecules in the confinement of the lipids may take a slightly different atomic structure than that determined from crystalline AMP [28].

## Supporting Information

Structure File S1 PDB structure files of the molecular structure in Figure 5 **D.**
(PDB)

Structure File S2 PDB structure files of the molecular structure in Figure 5 **E.**
(PDB)

Structure File S3 PDB structure files of the molecular structure in Figure 6.
(PDB)
